# Supplementary material for: Sea level extremes and compounding marine heatwaves in coastal Indonesia
Source: Nat Commun. 2022 Oct 27;13:6410. doi: 10.1038/s41467-022-34003-3 (PMC9613989; doi:10.1038/s41467-022-34003-3)
Supplement: Supplementary file 1 — Supplementary Information [file 41467_2022_34003_MOESM1_ESM.pdf]

Supplementary Tables

Year	1998	1998	2010	2013	2016	2016
**Month**	June	Nov	Dec	May	June	Dec
**SLA(m)**<br>Sat. (gauge)	0.23	0.26	0.31 (0.41)	0.28 (0.31)	0.44 (0.45)	0.35 (0.40)
**SSTA (ºC)**	1.45	1.25	1.11	1.26	1.81	1.21
**Climate Modes**	nIOD+<br>La Niña	nIOD+<br>La Niña	nIOD+<br>La Niña	nIOD<br>(early)	nIOD+<br>developing<br>La Niña	nIOD+<br>La Niña

Supporting Table 1a.

Year	1996	2008	2008	2012	2012	2013	2013	2015	2017
**Month**	Nov	Feb	Dec	Jan	Mar	Jan	Dec	Jan	Dec
**SLA(m)**<br>Sat.(gauge)	0.22	0.24<br>(0.27)	0.23<br>(0.27)	0.29<br>(0.36)	0.27<br>(0.34)	0.28<br>(0.34)	0.21<br>(0.26)	0.27<br>(0.30)	0.30
**Climate Modes**	nIOD	La Niña	La Niña	Mons	Mons	Mons	Mons	Mons	La Niña

Supporting Table 1b.

Supporting Table 1. Peak amplitude and corresponding time of the Compound Height-Heat EXtremes (CHHEXs; supporting Table 1a) and Height EXtremes (HEXs) alone (supporting Table 1b) during 1993-2018 identified from Fig 1, using the 90th percentile of monthly mean satellite sea level anomalies (SLA) of 0.21m as the threshold, together with satellite sea surface temperature anomalies (SSTA) near the Cilacap B tide gauge location at Java coast for CHHEX events, with the 90th percentile of SSTA (1.11ºC) as the threshold. Tide gauge SLA values are shown in parentheses. We choose to retain the mean seasonal cycle in the SLA and SSTA (see text for discussion), and the SSTA is relative to the 1989-2018 mean of 28.5ºC. Note that for the 2016 and 2010 CHHEX events the SSTA peaks lead the SLA peaks, but the SSTA remains above (close to) the 90th percentile during the SLA peak for 2016 (2010), and the SSTAs shown in supporting Table 1a are their peak values. Occurrence of the two dominant interannual climate modes over the Indo-Pacific Ocean, the El Niño-Southern Oscillation (ENSO) and Indian Ocean Dipole (IOD), are shown in the bottom row. El Niño (La Niña) is positive (negative) phase of ENSO, and nIOD (pIOD) denotes negative (positive) phase of IOD. “Mons” denotes either Indian monsoon or Australian-Indonesian monsoon. Note that the correlations for ENSO-Indian monsoon is -0.44 and for ENSO-Australian monsoon is -0.3 for 1993-2018, with both >95% significance.

Data (1993-2017)	Linear trend (mm/yr)	Correlation, r	STD (m)
**Satellite**	5.59±0.99	1.	0.12
**ORAS4**	3.97±1.00	0.98	0.13
**ROMS**	6.50±1.16	0.95	0.15
**HYCOM**	2.94±1.05	0.90	0.13
**Tide gauge (satellite) (2007-2016)**		0.99	0.17 (0.13)

Supporting Table 2. Linear trend & uncertainty from satellite sea level anomaly (SLA) data, ORAS4 reanalysis data (which is model simulation constrained by observed data), ocean general circulation model simulation from ROMS and HYCOM for the 1993-2017 period (2nd column). Correlation coefficient with satellite SLA (3rd column), standard deviation (STD) after removing linear trend (4th column). The last row shows correlation between tide gauge and satellite data (3rd column) and STD for tide gauge (satellite) SLA for the 03/2007-12/2016 period. All trends and correlation coefficients exceed 95% significance.

Supplementary Figures

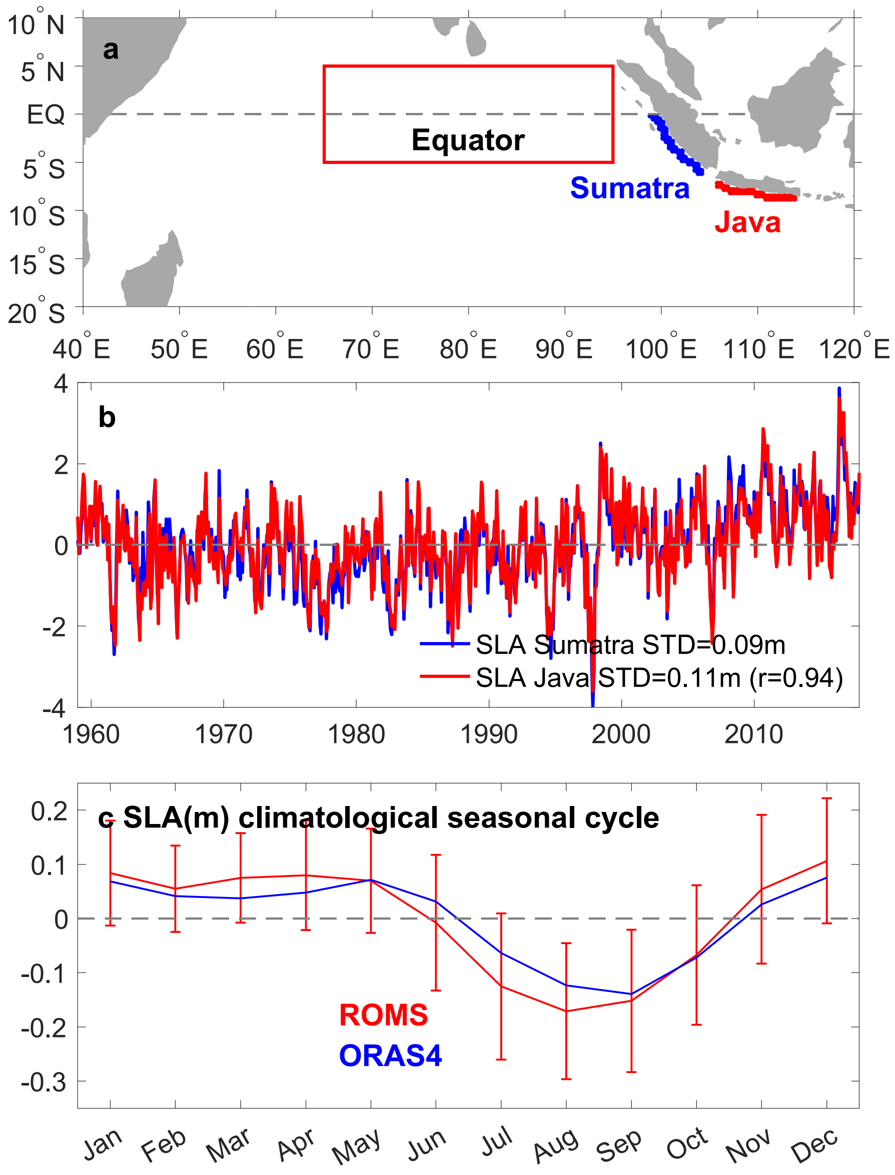

Supporting Figure 1. Regions and timeseries of area-averaged sea level anomalies (SLA). **a**, Regions where averaged equatorial zonal wind stress and longshore wind stress near coastal Sumatra and Java are calculated and are used as predictors of the Bayesian Dynamic Linear Model shown in Figure 4. **b**, Normalized SLAs (with mean seasonal cycle removed) from the Regional Ocean Modeling System (ROMS) simulation averaged over Sumatra and Java coastal areas. SLAs in the two regions are highly coherent (r=0.94) and obtain larger amplitude at Java coast, with standard deviation (STD) of 0.11m compared to 0.09m along the coast of southern Sumatra. **c**, Monthly SLA climatological seasonal cycle for the 1959-2017 period from ROMS (red) and ±1 STD of interannual variability (vertical line segments), together with monthly

climatology of ECMWF Operational Reanalysis System version 4 (ORAS4) SLA for 1959-2017 (blue).

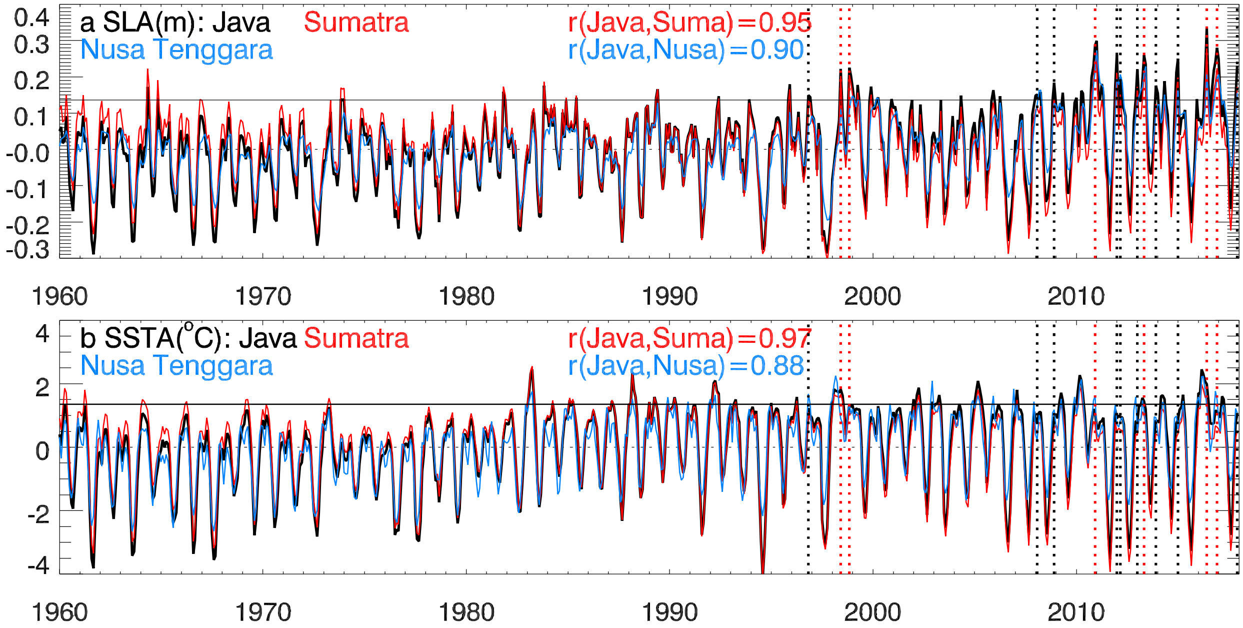

Supporting Fig 2. Time series of ORAS4 monthly mean sea level anomaly (SLA) and sea surface temperature anomaly (SSTA) averaged over Java coast (106°E-114°E, 7°S-9°S; black), Southern Sumatra coast (102°E-105°E,3°S-6°S; red), and east of Java along Nusa Tenggara coast (114°E-120°E, 8.5°S-10°S; blue) from 1960-2017. **a**, SLA; **b**, SSTA. The black horizontal line in each panel shows the 90th percentile of the black curve, which is area-mean SLA (in **a**) and SSTA (in **b)** along Java coast. Vertical dotted lines show the peak months of 15 HEX events with black lines indicating the nine Height Extreme (HEX)-alone events and red lines indicating the six Compound Height and Heat EXtreme (CHHEX) events identified in Fig 2a using satellite altimeter and SST data; these events are well captured by ORAS4 reanalysis data.

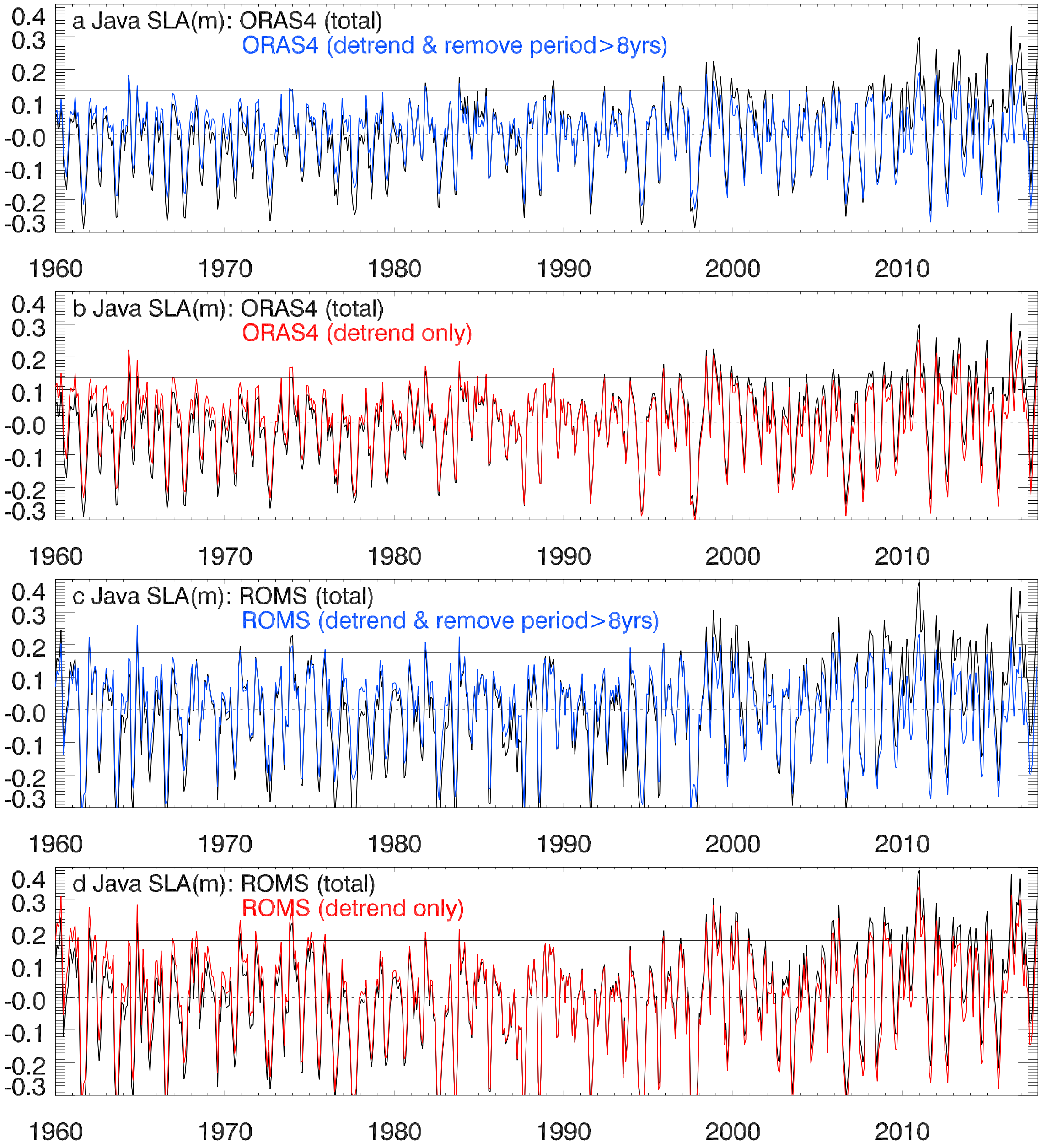

Supporting Figure 3**.** Time series of monthly mean sea level anomalies (SLAs) averaged over Java coastal area (106°E-114°E, 7°S-9°S; black). **a**, SLAs from ORAS4 reanalysis with the mean of 1960-2017 removed (black curve), and with linear trend plus decadal variability (8yr lowpass filtered using the Butterworth filter) removed (blue); the horizontal black line shows the 90th percentile of the black line; **b**, Same as **a** except for SLA that removes linear trend but retains decadal variability (red); **c**, Same as **a** but for ROMS model simulation; **d**, Same as **b** but for ROMS model simulation. The linear trend of 1960-2017 is used to represent global sea level rise effect.

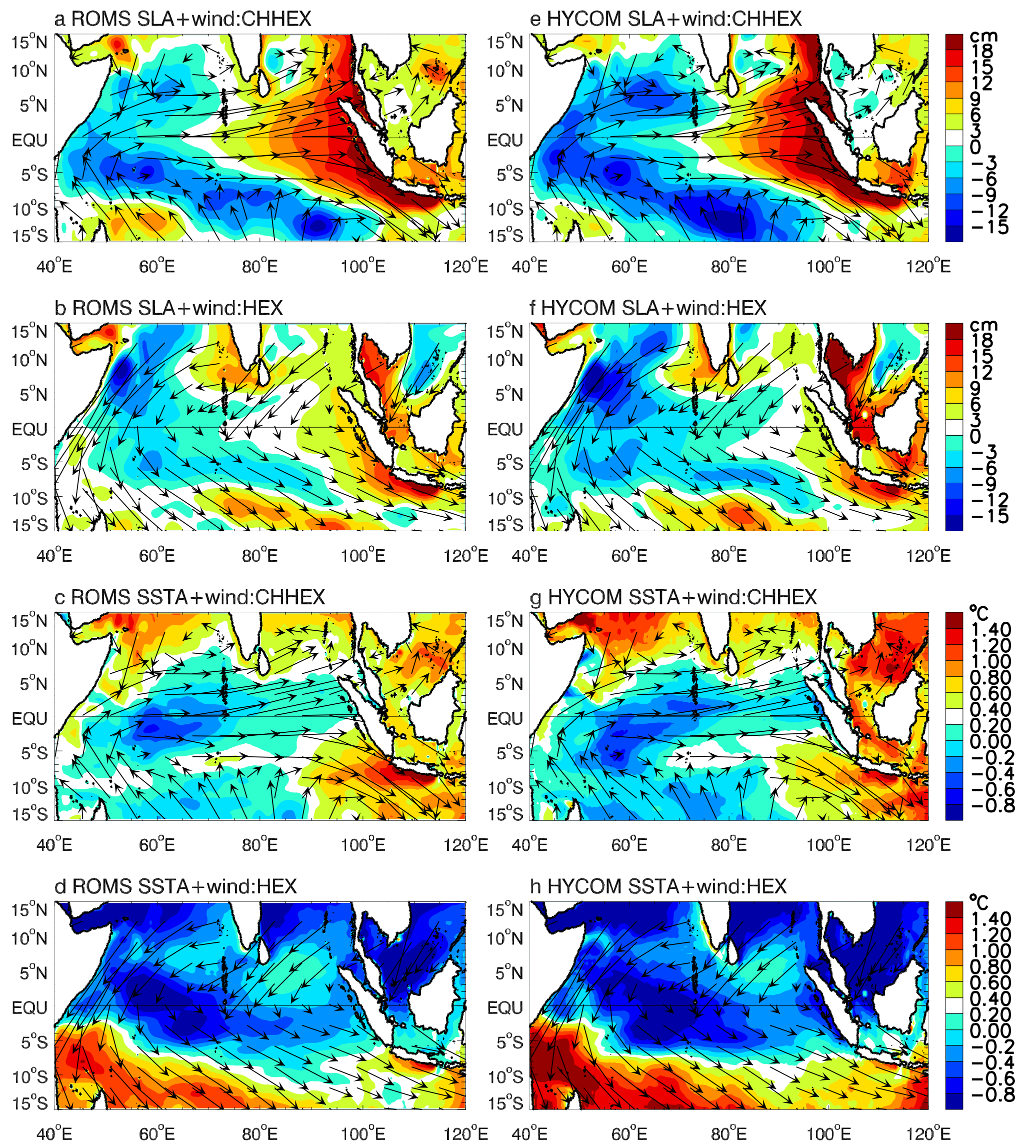

Supporting Figure 4. Composite maps of model simulated monthly SLAs, surface wind stress anomalies that are used to force ROMS and HYCOM, and SSTA for the peak months of the six CHHEX and nine HEX alone events. Panels **a-d** are the same as Figs **3a-3d** except for ROMS model simulations, with **a & b** representing SLA (color) and surface wind stress (arrows) for CHHEX & HEX alone events respectively and **c & d** representing SSTA (color) and surface wind stress (arrows) for the CHHEX & HEX alone events respectively. Panels **e-f** are the same as **a-d** except for HYCOM model simulations.

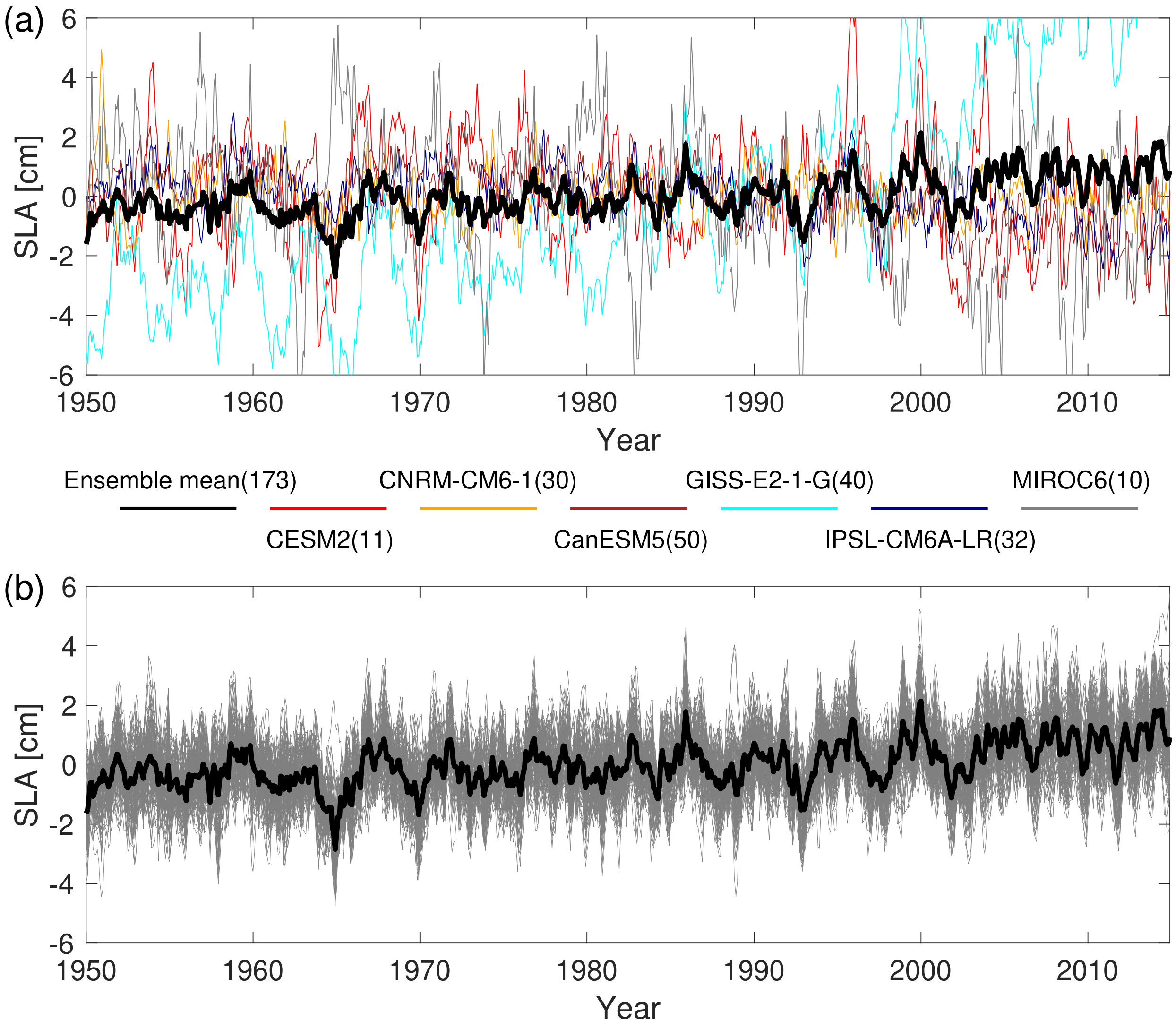

Supporting Figure 5. Externally forced dynamical sea level anomalies (with global mean sea level removed) averaged for Java coastal area (106˚E-114˚E; 10˚S-8˚S) from the large-ensemble experiments of seven models that participated in the Coupled Model Intercomparison Project Phase 6 (CMIP6) being assessed by the Intergovernmental Panel on Climate Change Sixth Assessment Report (IPCC AR6). **a**. The 173-member ensemble mean (black) and ensemble means from individual models with ensemble members $\geqslant$10 from each model (color). **b**. The 173-member ensemble mean as in **a** (black) and its uncertainty spread estimated using Monte-Carlo experiment (gray lines)[1]. We randomly pick 40 ensemble members from the 173 and repeat for 100 times.

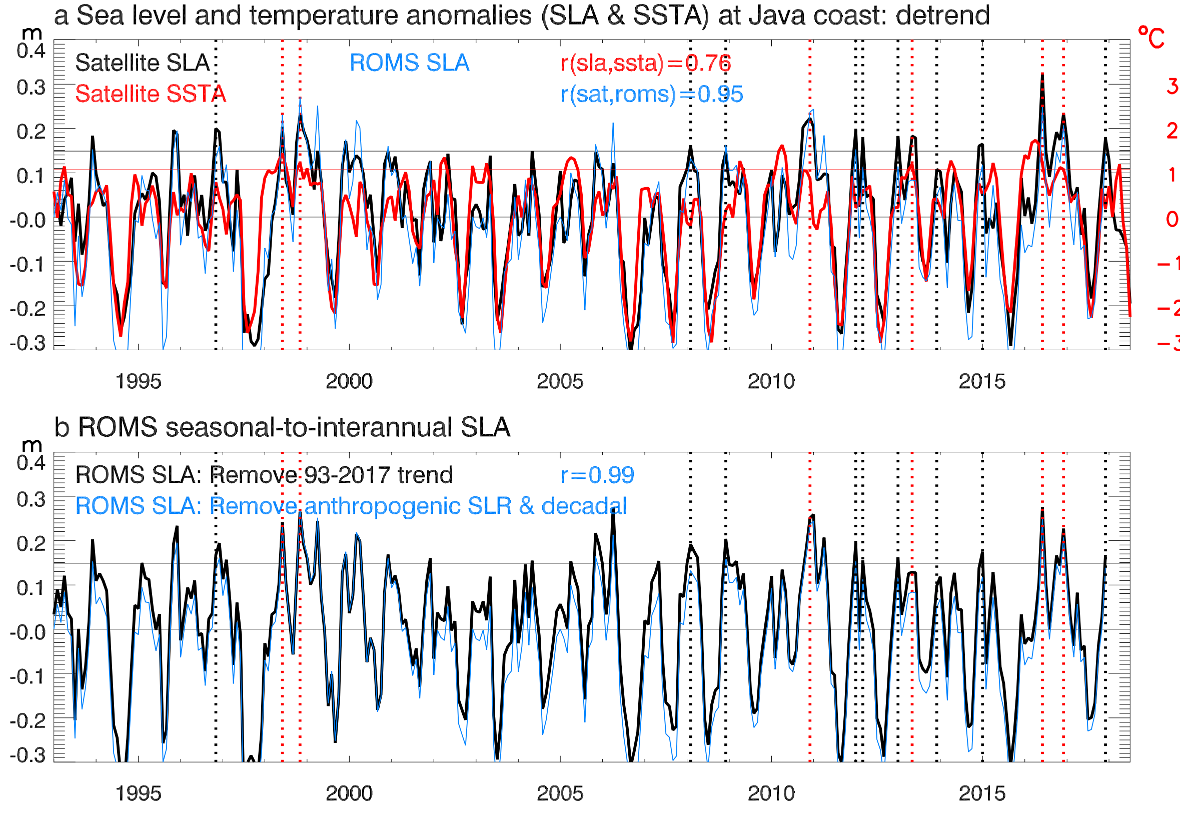

Supporting Figure 6. Time series of monthly SLA and SSTA near the Java tide gauge location (circle in Figs 1b-1c) from satellite observations and ROMS model simulation. **a,** Satellite SLA (black) and SSTA (red) with their linear trends of 1993-2018 removed, together with ROMS SLA with 1993-2017 trend removed (blue). The correlation coefficient for satellite SLA & SSTA is 0.76, and for satellite SLA & ROMS SLA is 0.95, both > 95% significance. Note that the Dec 2013 event falls below the 90th percentile. **b**, ROMS SLA with 1993-2017 trend removed (black, same as blue in **a**), and ROMS SLA by first removing anthropogenic global sea level rise and then remove 8yr lowpass filtered SLA from 1960-2017 but only the 1993-2017 period is shown (blue), which isolates seasonal-to-interannual anomalies. The high correlation (r=0.99) between the two curves suggests that by removing the short-term trend of satellite observation period, we essentially remove global sea level rise and a large portion of decadal variability; therefore, the detrended SLA and SSTA shown in this figure primarily represent seasonal-to-interannual anomalies.

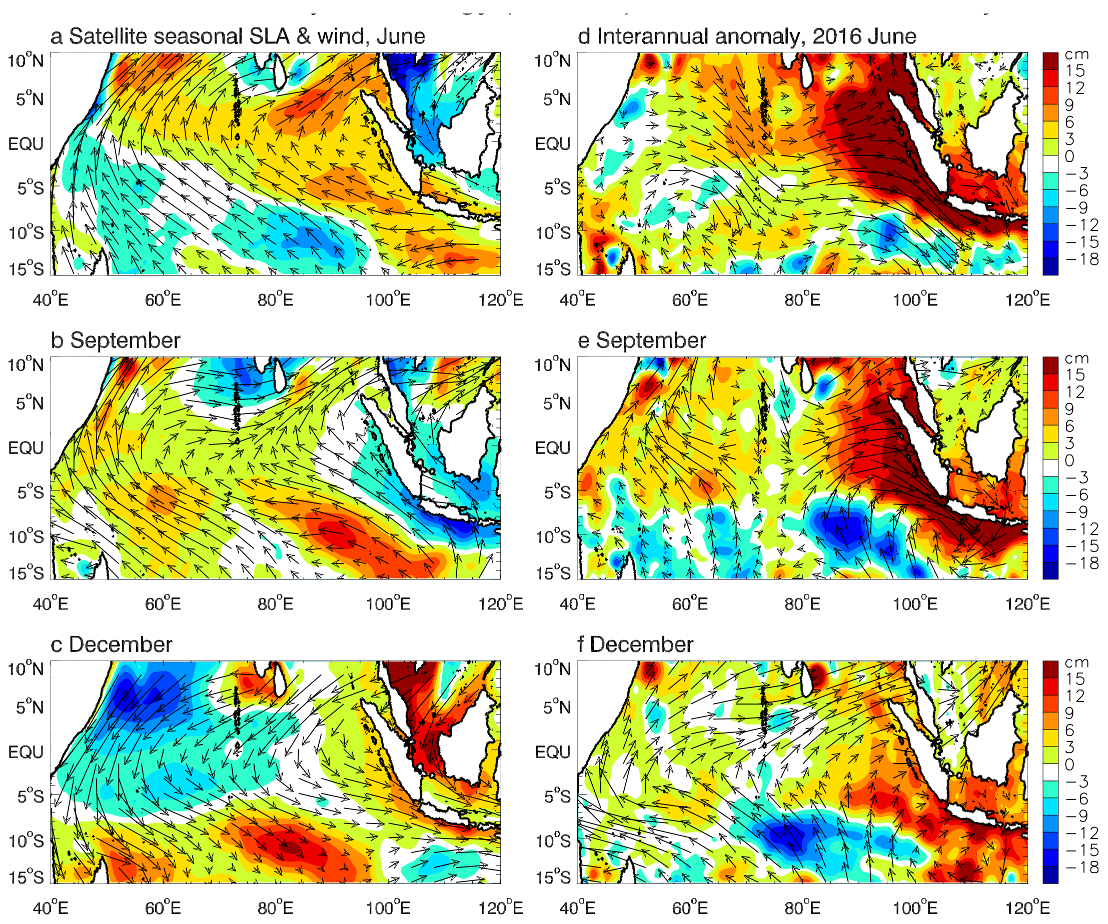

Supporting Figure 7. Satellite observed monthly mean sea level and CCMP surface wind anomalies for June, September and December. **a-c,** The mean seasonal cycle for the 1993-2018 period (with 1993-2018 mean removed); **d-f**, Interannual anomalies of 2016 (with mean and seasonal cycle removed).

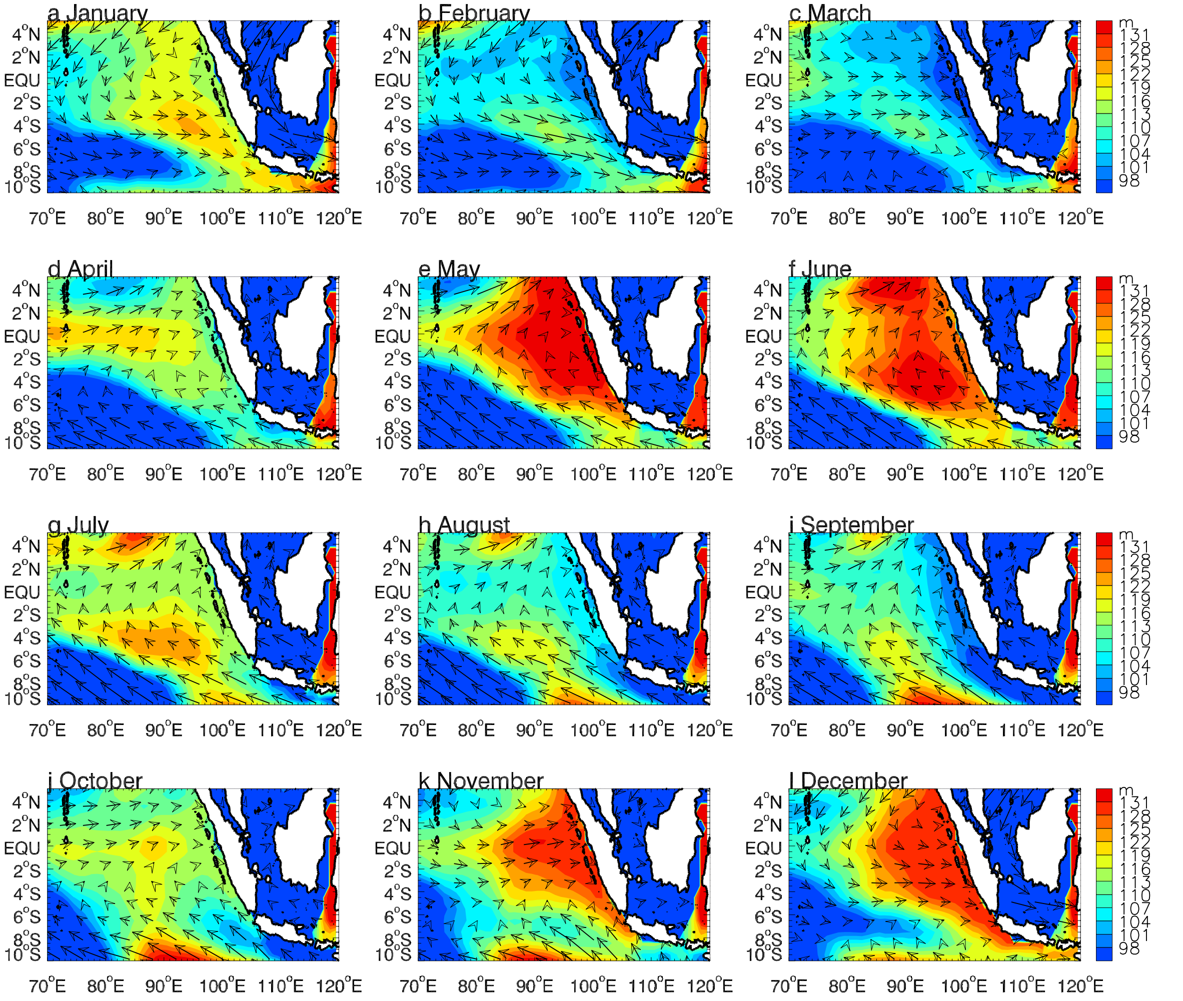

Supporting Figure 8. Monthly mean climatology of JRA55-do reanalysis surface wind stress (arrows) and thermocline depth, measured by the depth of 20°C isotherm (color), from ORAS4 reanalysis for the 1988-2017 30yr period.

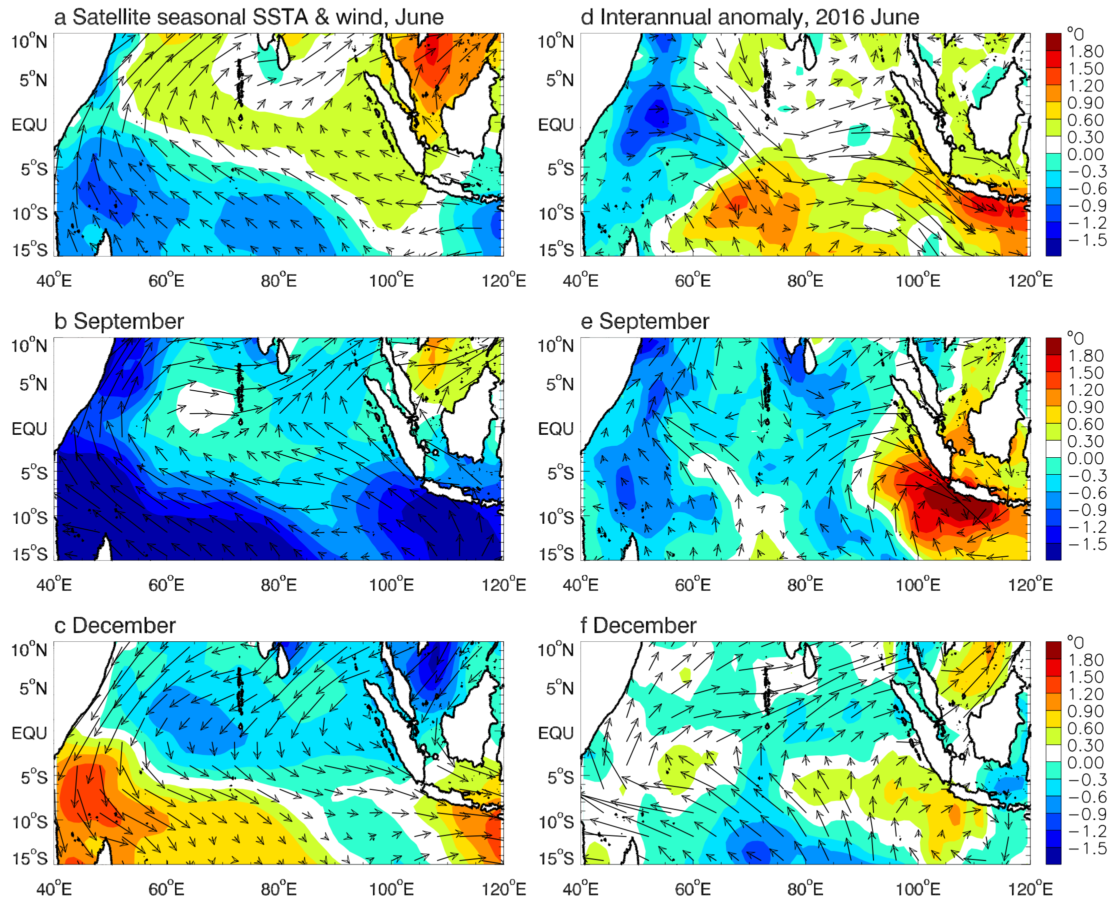

Supporting Figure 9. Satellite observed monthly mean sea surface temperature (SST) and surface wind stress anomalies based on the 1993-2018 period for June, September and December. **a-c,** The mean seasonal cycle of 1993-2018 period (with 1993-2018 mean removed); **d-f**, Interannual anomalies of SST and surface winds for 2016 (with 1993-2018 mean and seasonal cycle removed).

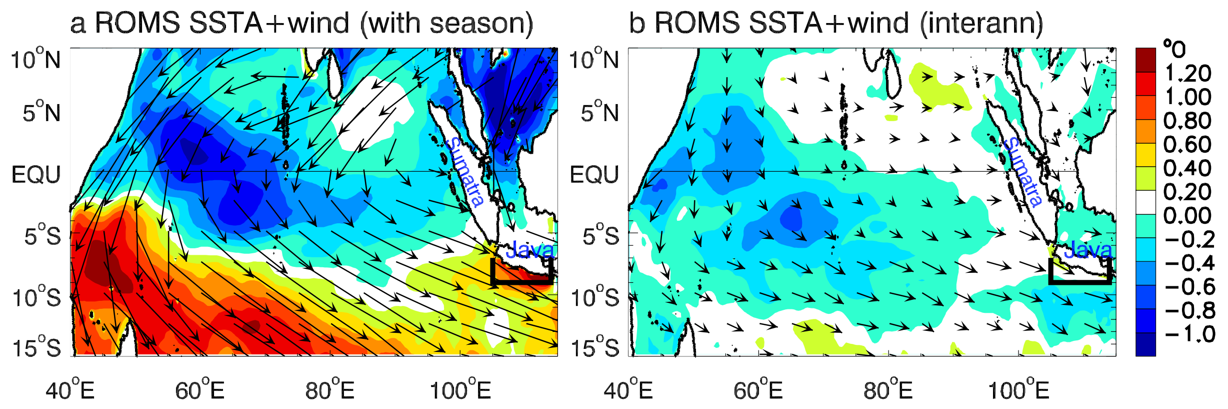

Supporting Figure 10. Composite of ROMS simulated monthly sea surface temperature anomalies (SSTA) and surface wind stress anomalies for the peak months of nine HEX alone extreme events. **a**, SSTA and wind with seasonal cycle included and **b**, with seasonal cycle removed and thus represent interannual variability.

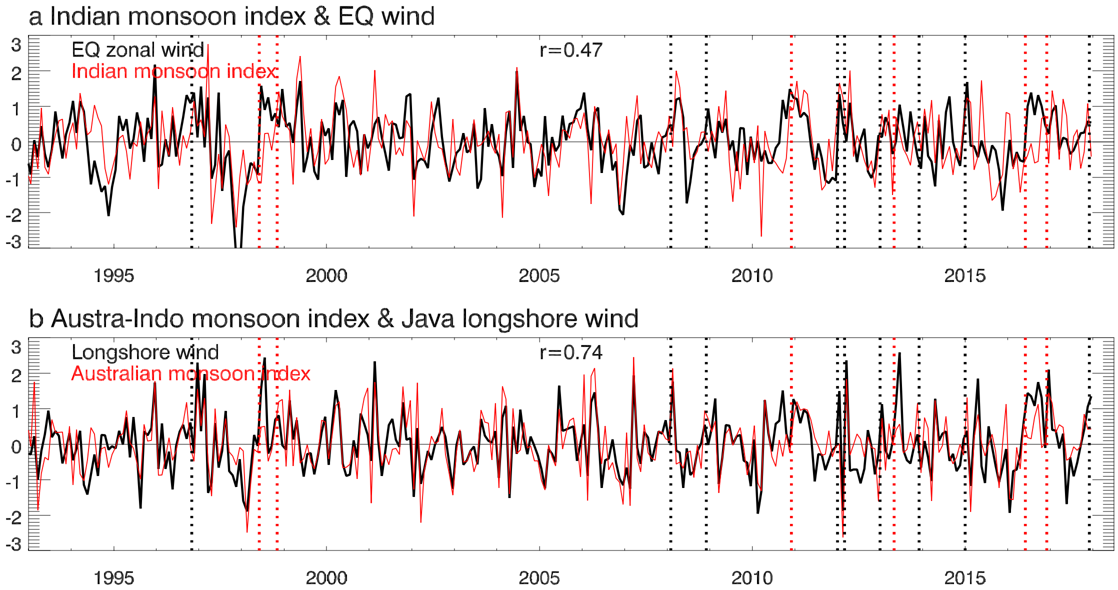

Supporting Figure 11: **a**, Time series of monthly mean equatorial zonal wind stress anomalies averaged over (65°E-95°E, 5°S-5°N, red box of supporting Fig 1a) from JRA55-do reanalysis data (black) and Indian monsoon wind shear index (red); both are taken as one month lead of sea level anomalies. **b**, The same as **a** but for Java longshore wind (averaged over coastal Java area shown in supporting Fig 1a) and Australian monsoon index. Mean seasonal cycle and decadal variability including trend (8yr lowpassed) for the 1960-2017 period are removed from each time series. Correlations shown are for 1960-2017 period (> 99% significance) but only 1993-2017 is shown for visibility. The vertical-dotted lines show the peak month of six CHHEX events (red) and nine HEX alone events (black).

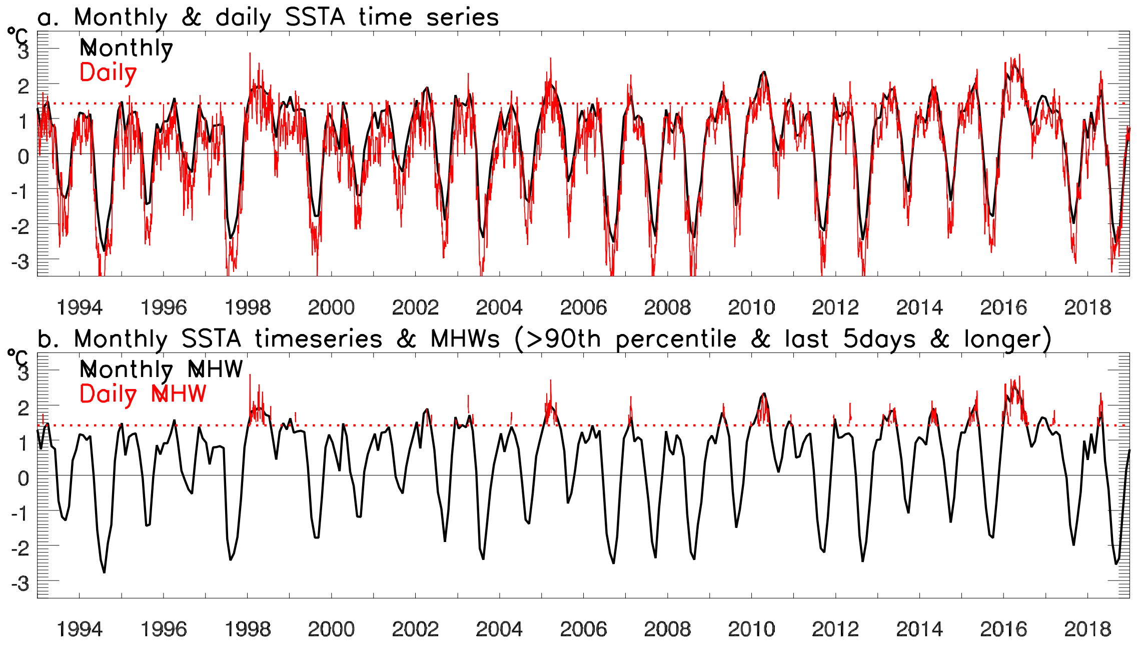

Supporting Figure 12. Time series of monthly and daily SSTA relative to the same 30-year mean of daily SST from 1989-2018, and marine heatwaves (MHWs) defined by both the monthly and daily SSTA. **a,** Monthly (black) and daily (red) time series of SSTA averaged over Java coastal area of (106°E-114°E, 7°S-9°S), the same region as in supporting Figs 2 and 3; the horizontal dotted red line represents the 90th percentile of daily SSTA. **b**, Time series of monthly SSTA (black; same as that in **a**) with the SSTA values exceeding the 90th percentile defined as MHWs, and the MHWs identified from daily data (red), which are defined as discrete prolonged anomalously warm water events when daily SSTAs exceed the 90th percentile and persist for at least 5 days. Note that the monthly SST data are on 1°x1^{o} grid points and daily SST data are on 0.25^{o}x0.25^{o} grid points.
